# Understanding Physician’s Perspectives on AI in Health Care: Protocol for a Sequential Multiple Assignment Randomized Vignette Study

**DOI:** 10.2196/54787

**Published:** 2024-04-04

**Authors:** Jane Paik Kim, Hyun-Joon Yang, Bohye Kim, Katie Ryan, Laura Weiss Roberts

**Affiliations:** 1 Department of Psychiatry and Behavioral Sciences Stanford University School of Medicine Stanford, CA United States

**Keywords:** AI-based clinical decision support, decision-making, hypothetical vignettes, physician perspective, web-based survey, hypothesis-driven research, ethics, stakeholder attitudes

## Abstract

**Background:**

As the availability and performance of artificial intelligence (AI)–based clinical decision support (CDS) systems improve, physicians and other care providers poised to be on the front lines will be increasingly tasked with using these tools in patient care and incorporating their outputs into clinical decision-making processes. Vignette studies provide a means to explore emerging hypotheses regarding how context-specific factors, such as clinical risk, the amount of information provided about the AI, and the AI result, may impact physician acceptance and use of AI-based CDS tools. To best anticipate how such factors influence the decision-making of frontline physicians in clinical scenarios involving AI decision-support tools, hypothesis-driven research is needed that enables scenario testing before the implementation and deployment of these tools.

**Objective:**

This study’s objectives are to (1) design an original, web-based vignette-based survey that features hypothetical scenarios based on emerging or real-world applications of AI-based CDS systems that will vary systematically by features related to clinical risk, the amount of information provided about the AI, and the AI result; and (2) test and determine causal effects of specific factors on the judgments and perceptions salient to physicians’ clinical decision-making.

**Methods:**

US-based physicians with specialties in family or internal medicine will be recruited through email and mail (target n=420). Through a web-based survey, participants will be randomized to a 3-part “sequential multiple assignment randomization trial (SMART) vignette” detailing a hypothetical clinical scenario involving an AI decision support tool. The SMART vignette design is similar to the SMART design but adapted to a survey design. Each respondent will be randomly assigned to 1 of the possible vignette variations of the factors we are testing at each stage, which include the level of clinical risk, the amount of information provided about the AI, and the certainty of the AI output. Respondents will be given questions regarding their hypothetical decision-making in response to the hypothetical scenarios.

**Results:**

The study is currently in progress and data collection is anticipated to be completed in 2024.

**Conclusions:**

The web-based vignette study will provide information on how contextual factors such as clinical risk, the amount of information provided about an AI tool, and the AI result influence physicians’ reactions to hypothetical scenarios that are based on emerging applications of AI in frontline health care settings. Our newly proposed “SMART vignette” design offers several benefits not afforded by the extensively used traditional vignette design, due to the 2 aforementioned features. These advantages are (1) increased validity of analyses targeted at understanding the impact of a factor on the decision outcome, given previous outcomes and other contextual factors; and (2) balanced sample sizes across groups. This study will generate a better understanding of physician decision-making within this context.

**International Registered Report Identifier (IRRID):**

DERR1-10.2196/54787

## Introduction

The implementation of artificial intelligence (AI) in health care is recognized as a promising means to advance medicine by providing timelier diagnoses, reducing administrative burden, and predicting outcomes with higher accuracy. As the availability and performance of AI-based clinical decision support (CDS) systems improve, physicians and other care providers poised to be on the front lines will be increasingly tasked with using these tools in patient care and incorporating their outputs into clinical decision-making processes. Due to the few instances of CDS systems in current use by frontline physicians, there is limited empirical research studying how these tools and the specific characteristics of these tools influence physicians’ engagement with AI and their clinical decision-making.

Previous research has identified factors that may influence physicians’ use and decision-making in the context of AI-based CDS systems, including clinical risk, explainability, as well as trust and transparency [1,2]. While the role of trust and transparency in clinician acceptance has been emphasized in the literature in recent years, findings supporting the claim that transparency about AI can actually support clinical decision-making are mixed [3-5]. Jussupow et al [6] used vignette-based experiments with physicians to understand how AI influences their decision-making, finding that the timing of the presentation of the AI result, whether or not the AI result aligns with the physician’s opinion, and the experience level of the physician may all be factors that influence the decision-making of a physician who is tasked with incorporating an AI-based CDS system in their diagnostic care. Such knowledge is critical to ensuring successful implementation of AI in health care, but due to the many differences in the types of applications of AI, subspecialties, and health care systems, there is a high degree of heterogeneity in physician attitudes regarding acceptance, and factors influential to their decision-making are likely to be context specific.

Experimental vignette studies such as Jussupow et al [6] provide an effective means to explore emerging hypotheses regarding how context-specific factors may impact physician acceptance and use of AI-based CDS tools or to generate new hypotheses regarding how individuals may react to specific scenarios involving new applications where much is unknown. Vignettes are narrative representations of real-world scenarios, providing context-specific stimuli and characteristics with which respondents may practice decision-making without actual exposure to such scenarios [7-9]. Experimental vignette studies have been used to understand reactions toward algorithmic errors [10], physician liability while using AI [11], and physician diagnostic accuracy in the context of AI [12].

In this study, we use a novel vignette survey design—“sequential multiple assignment randomization trial (SMART) vignettes,” proposed by JP Kim and HJ Yang (unpublished data)—which uses the SMART design developed by Murphy [13] in a web-based vignette survey. The novelty of this survey design is that it features 2 design elements previously not seen in conventional designs: sequential randomization and adaptive allocation. In experimental vignette studies, hypothetical characteristics (factors) are varied according to levels. Each respondent is randomly assigned to 1 vignette characterized by a specific realization of each factor in order to test how the factors influence responses. In conventional vignette studies, this randomization typically occurs once before the study is administered at baseline, followed by a complete set of questions. Sequential randomization, on the other hand, involves randomly assigning respondents to vignettes multiple times throughout the survey, with each randomization point followed by a subset of questions pertaining to the factor being tested. This latter approach allows inferences about the final response based on previous factors and responses. In contrast, baseline randomization in traditional designs is limited to inferences on how likely each combination of responses is based on given factors. Adaptive allocation is a way to adjust randomization probabilities to encourage more balanced groups for such inference. While these 2 concepts are not novel in and of themselves—the former appears in the field of SMARTs [13,14] and the latter is from Efron [15]—the application of such concepts in the context of vignette survey methodology is novel, to the best of our knowledge.

To best anticipate the decision-making of frontline physicians in scenarios involving AI CDS tools, a hypothesis-driven approach that enables scenario testing in advance of clinical implementation of these tools is urgently needed. As such, we propose to undertake an empirical study engaging frontline physicians in order to better understand the causal effects of specific contextual and algorithmic features on their clinical judgments and perspectives. For our approach, we will conduct a “SMART vignette” web-based survey that features hypothetical scenarios based on emerging or real-world applications of AI-based CDS systems. These scenarios will vary systematically by features related to clinical risk, the amount of information provided about the AI, and the AI result in order to test and determine the causal effects of specific factors on the judgments and perceptions salient to physicians’ clinical decision-making.

## Methods

### Overview

The purpose of this study is to better understand the impact of algorithm-related features on physician acceptance and attitudes related to clinical decision-making in the context of AI CDS tools. US-based physicians with specialties in the family or internal medicine (ie, those most poised to be on the “front lines” of nonemergency patient care) who are listed in the most recent version of the American Medical Association (AMA) Physician Masterfile (PMF) will be recruited through email and mail (target n=420). Through a web-based survey, participants will respond to baseline questionnaires regarding their demographics, professional experience, and attitudes toward and experience with AI or machine learning (ML) in medicine. They will then be randomly assigned to a 3-part “SMART vignette” detailing a hypothetical clinical scenario involving an AI decision support tool. Each respondent will progress through the multistage vignette survey, and at each stage, they will be randomly assigned to one of the possible vignette variations. The 3 randomization points will vary in regard to the level of clinical risk (higher risk vs lower risk), the amount of information provided about the AI (more information vs less information), and the certainty of the AI output (higher certainty vs lower certainty). After each randomization point, participants will be asked to respond to questions regarding their hypothetical decision-making as it relates to these factors.

This study is part of a broader project studying stakeholder views regarding the development and use of AI and ML in medicine (National Center for Advancing Translational Sciences R01-TR-003505). It is built upon findings from the first phase of the broader project, in which AI and ML researchers and physicians were interviewed regarding ethical considerations they have encountered or anticipated in the development, refinement, and application of AI and ML in medicine [16,17]. Insights from this phase of research were used to create hypothetical but realistic scenarios for this study and to inform the development of questions assessing physician decision-making in these scenarios.

### Recruitment

US-based physicians who are listed in the most recent version of the AMA PMF as specializing in family medicine or internal medicine will be eligible to participate in this study. The PMF is a comprehensive database of US physicians used for verifying professional credentials. Through the AMA-approved third-party vendor Medical Marketing Services, Inc, we will obtain the email and mailing addresses of a sampling frame of 10,000 physicians who meet our inclusion criteria. Physicians who are identified in this sampling frame will be contacted with invitations to participate in our web-based survey.

Through the third-party vendor Medical Marketing Services, Inc, we will send up to 4 email invitations to each physician in the sampling frame. Each email will provide a short description of our study and a web link to the web-based survey. Based on previous studies of this method of recruitment, we anticipate a 1.5% response rate [18]. If the target recruitment goal of 420 is not reached after 4 email invitations are sent, we will send a follow-up invitation by mail to any physicians in the original sampling frame who have not yet responded to the survey. Each mailed invitation will briefly describe our study and will contain a web address for the web-based survey.

### Ethical Considerations

This project has received human participant research ethics approval from the Stanford University Institutional Review Board (65168). Upon navigating to the web-based survey, potential participants will be presented with a web-based informed consent form that details the content of the survey and the anticipated risks and benefits of participation. Participants will only proceed to the web-based vignette survey if they provide consent. Participation will be voluntary, and all responses will be anonymous. All participants will be compensated for their time and effort with a US $10 Amazon gift code at the completion of the survey.

### Hypotheses and Outcome Variables

Our hypotheses are the following:

1. Hypothesis 1: higher degrees of clinical risk associated with the algorithm will influence lower levels of physician agreement with the effectiveness of and confidence in using the algorithm to help guide treatment decisions.
2. Hypothesis 2: disclosure of details of the algorithm will impact physicians’ attitudes of confidence and efficacy, depending on their previous exposure to AI (ie, education, training, and clinical experience). We hypothesize that greater disclosure will influence positive attitudes among physicians with greater exposure but will have a negative or no impact on physicians with less exposure.

The primary outcome variables will be physicians’ ratings, on a Likert scale, of perceived confidence in the AI algorithm in helping the physician make the best treatment recommendation for their patient (eg, “The AI result improved the confidence I have in my final decision”; from 1=“strongly disagree” to 5=“strongly agree”), as well as the perceived effectiveness of the use of the AI algorithm (eg, “Overall, the AI improved the care that I was able to provide to the patient”; rated on a 5-point scale from 1=“strongly disagree” to 5=“strongly agree”).

### Survey Design

#### Survey Instruments

Demographic characteristics (age, gender, educational level, race, and ethnicity) and professional characteristics (specialty type and years of experience) will be assessed through a baseline demographics questionnaire (13 questions). Experience with and attitudes toward AI and ML applications in medicine will be assessed using adapted measures (7-10 questions assessing experience and 11 questions assessing attitudes) [19,20]. A 3-part sequentially randomized vignette detailing a clinical scenario involving a CDS system will be presented to each participant; follow-up questions will assess participant attitudes and decision-making in the context of the vignette scenario (21 questions).

#### Vignette Development and Design

The vignettes used in this study were developed based on scenarios encountered by researchers and clinicians, as told in interviews in the first phase of this project [16]. Based on these findings, the dimensions in which the vignettes are systematically varied include the context of the decision-making scenario (higher risk vs lower risk), the amount of information provided regarding the algorithm (more information vs less information), and the certainty of the AI output (higher certainty vs lower certainty). The full content of the vignettes is available in Multimedia Appendix 1.

#### SMART Design

We have harnessed a design element that originates from clinical trial design: SMART [13,21,22]. JP Kim and HJ Yang (unpublished data) report the design. First, we use randomization probabilities to assign the subsequent vignette, which allows for tailoring the sequence of assigned vignettes to the individual instead of predetermined assignments of combinations of vignettes. The web-based survey format is particularly amenable to the design in which the subsequent questions are randomized. To the best of our knowledge, the SMART design has not yet been applied in the area of survey designs. SMART designs have been used in the evaluation of treatment algorithms for several psychiatric disorders (eg, depression in the Sequenced Treatment Alternatives to Relieve Depression and Clinical Antipsychotic Trials of Intervention Effectiveness studies) and for the treatment of advanced prostate cancer [23,24].

**Figure 1:**
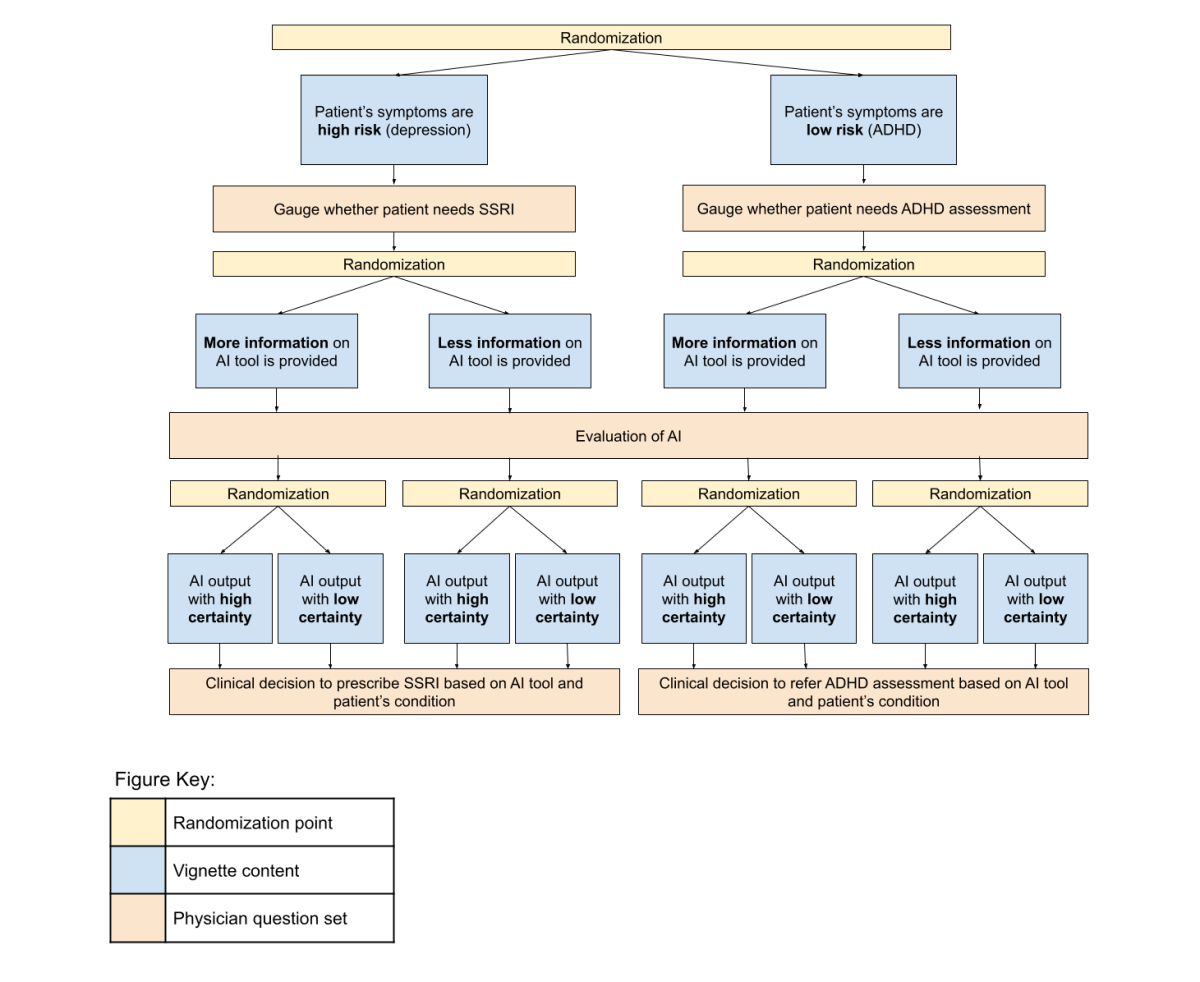
Study design for the web-based vignette survey featuring sequential randomization. Yellow boxes indicate a randomization point, blue boxes indicate vignette content, and orange boxes indicate responses. Respondents will be physicians in family medicine or internal medicine across the United States. ADHD: attention-deficit/hyperactivity disorder; AI: artificial intelligence; SSRI: selective serotonin reuptake inhibitors.

As shown by Figure 1 (adapted from JP Kim and HJ Yang—unpublished data), we configured our SMART vignette to have 3 dimensions, each with 2 levels: patient risk (higher risk vs lower risk), information on AI (more information vs less information), and certainty of AI output (higher certainty vs lower certainty), respectively. Corresponding vignette scenarios and question sets were input for each combination of randomization sequences. One question from every question set was flagged to serve as a primary question, the response for which was used to adaptively allocate the participants [16,17]. For 5-point Likert scale primary questions, participants who respond from “1” to “3” are assigned to response group 0, while those who respond with “4” to “5” are assigned to response group 1. For yes or no questions, “yes” corresponds to response group 1 and “no” to response group 0. The probability of Efron’s [15] biased coin was prespecified to be 0.667.

### Power Analysis

We performed a sample size estimation for determining the best embedded dynamic treatment regime using the approach outlined in Artman et al [25] for power analysis in a SMART design. Using the R package *smartsizer* (R Foundation for Statistical Computing) that implements Monte Carlo simulations in Artman et al [25], we calculated that the number of individuals needed to enroll in the vignette experiment is 420 to guarantee at least 80% power to detect an effect size of 0.15.

### Survey Implementation

The SMART vignette survey will be administered on the internet using a web application created by our research team and hosted by Stanford University [26]. The application was developed using Python Flask and deployed using Amazon Beanstalk and Elastic File System, with survey responses stored on a secure SQLite database. This platform allows sequential randomization and adaptive allocation, which are features not yet offered by other survey software such as Qualtrics or Research Electronic Data Capture (REDCap; Vanderbilt University).

The study survey went through a round of internal testing before its launch. Dummy responses were created to ensure data were being properly parsed and stored. The research team tested the application on various devices to identify errors and provide feedback on the user experience and user interface. After a few rounds of internal testing, all study responses and parameters were reset for launch.

Unique user IDs were generated for each potential participant. As part of the recruitment emails and letters, participants will be asked to log into the SMART vignette survey using their assigned user ID and a provided study ID. Respondents have the option to exit and return to the survey at any time. Once a respondent has submitted a complete response, a US $10 Amazon gift card code will be claimed and displayed. The study will remain open to responses until the target number of responses is reached.

### Statistical Analysis

Regression models will be used to determine the causal effects of contextual dimensions on clinician judgments, where confidence is an outcome and the assignment indicator, age, gender, and race are variables. We will estimate “conditional vignette effects” on physicians’ judgments (ie, the effect of the vignette context within subgroups defined by strata of the baseline variables).

## Results

This study was funded in September 2020 by the National Center for Advancing Translational Sciences. After the completion of internal testing, the survey was officially launched on May 16, 2023. Email recruitment occurred between May 16 and August 18 and resulted in 35 complete responses. Mail recruitment began on October 1, 2023, and will continue until the target number of 420 participants is reached. It is anticipated that we will reach this number by 2024. The survey will be closed, and data analysis will begin immediately after the target number of responses is reached.

## Discussion

As AI technologies in medicine continue to advance, AI-based CDS tools are expected to be increasingly integrated into frontline care, and physicians will be faced with incorporating these tools to inform the evaluation and management of large numbers of patients with diverse needs. This web-based SMART vignette study will generate a better understanding of frontline physician decision-making in the context of AI-based CDS systems and will provide information on how specific factors, such as the level of clinical risk, the amount of information provided to the physician about the AI, and the certainty of the AI output, may influence physician decision-making and related attitudes.

The clinical and algorithmic factors tested in this project will expand on insights from previous research. In at least 1 previous study, risk perception was indicated to influence physicians’ willingness to adopt AI-based tools in clinical practice [27]. By testing this factor in controlled scenarios involving moderately varied levels of clinical risk, our study will offer the opportunity to fine-tune our understanding regarding the effect of risk on physician decision-making involving AI. Additionally, while previous studies have demonstrated physician’s preferences for greater explainability, this has not had a demonstrated effect on their overall clinical decision-making [1]. As a possible alternative to explainable AI, our study will examine whether simply providing more information about an AI affects frontline physicians’ attitudes and their related decision-making. Finally, the analysis of physician decision-making in the context of varied AI output certainties will expand on Jussupow et al [6] by allowing us to identify the individual and context-specific factors that may contribute to a physician’s overall acceptance of an AI output.

To empirically test these factors, this study uses a novel vignette study method, “SMART vignettes,” an adaptation of the SMART design developed by Murphy [13], applied to web-based survey designs. Further details on the methodology can be found in JP Kim and HJ Yang (unpublished data). This novel method leverages 2 new design characteristics, namely sequential randomization and adaptive allocation. Our newly proposed “SMART vignette” design offers several benefits not afforded by the extensively used traditional vignette design, due to the 2 aforementioned features. These advantages are (1) increased validity of analyses targeted at understanding the impact of a factor on the decision outcome, given previous outcomes and other contextual factors; and (2) balanced sample sizes across groups.

Strengths of the proposed survey include the use of the sequential randomization design feature, which will allow the presentation of vignettes to be tailored to the individual. Our design offers an advantage above a “rule-based, branching logic approach,” the predominant approach used in web-based surveys, in which a particular response determines the subsequent question to be the same across all respondents [28,29], as well as a single-time baseline randomization in conventional vignette studies. The use of sequential randomization in this web-based survey will enable assessments of the causal effects of contextual information in the algorithms on the judgments of physicians entrusted with applying and implementing the use of the algorithms. In addition, the adaptive quality of the survey may allow for a greater sense of interactivity from the perspective of the survey respondent. Potential limitations of this study include a low response rate, as previously documented in the literature regarding physicians, as well as possible response fatigue, as is commonly experienced in survey studies. Another limitation is that the novel design features we presented are not yet widely available on existing survey platforms (eg, REDCap and Qualtrics) and thus may limit the reproducibility of this particular method. This represents an area of future research.

AI-augmented tools have the potential to improve physicians’ decision-making and productivity by automating referrals and triage, augmenting in-person and between-visit treatment, and assisting with minimally invasive procedures. Given this potential, there is a need to better anticipate decision-making in AI-augmented settings as well as identify potential vulnerabilities that may compromise decision-making [30]. Our proposed study comes from the approach of “stakeholder research,” the work of seeking perspectives from individuals impacted by the situation at hand, and fills a missing gap in the literature. As Rahwan et al [31] noted in their study, “machine behavior...cannot be fully understood without the integrated study of algorithms and the social environments in which algorithms operate.” Such work is needed to understand the range of physician decisions and the causal impact of attributes of AI-embedded care, particularly in high-stakes settings, and vignettes are well suited to address this need.

## Appendix

Multimedia Appendix 1 Vignettes.

## Abbreviations

AI: artificial intelligence
AMA: American Medical Association
CDS: clinical decision support
ML: machine learning
PMF: Physician Masterfile
REDCap: Research Electronic Data Capture
SMART: sequential multiple assignment randomization trial

## Data Availability

Data sharing is not applicable to this article as no data sets were generated or analyzed during this study.
