# Supplementary material for: Understanding Physician’s Perspectives on AI in Health Care: Protocol for a Sequential Multiple Assignment Randomized Vignette Study
Source: JMIR Res Protoc. 2024 Apr 4;13:e54787. doi: 10.2196/54787 (PMC11027055; doi:10.2196/54787)
Supplement: Multimedia Appendix 1 [file resprot_v13i1e54787_app1.docx]

**RANDOMIZATION**

↙ ↘

**Vignette Path #1** (see below) **Vignette Path #2** (see page 3)

**Vignette Path #1**

| **Degree of clinical risk - HIGHER** |
| --- |
| Jess Y. is a 33-year-old white female who presents to your clinic. She reports feeling extremely down and out of sorts over the past three months.  Jess reports feeling stressed about her full-time job as a software engineer at a company where she has worked for six years. Although she is interested in the work, Jess reports recently experiencing feelings that it is pointless or not worthwhile. She reports feeling tired and unfocused at work, despite getting 8-9 hours of sleep a night.  Jess also reports increasing tension between her and her partner of five years. Previously, they would spend their evenings and weekends together going on hikes, riding bikes, or meeting up with friends. Recently however, Jess hasn’t had the energy to participate in these outings and instead stays home to watch TV or simply “lay on the couch and do nothing.”  Jess reports that she attended therapy for several years during college for feelings of depression, but that she did not find the therapy particularly effective. |

↓

**RANDOMIZATION**

↙ ↘

| **Amount of information sharing - LOWER** | **Amount of information sharing - HIGHER** |
| --- | --- |
| Recently, the hospital system you work for has adopted routine use of an artificial intelligence tool called *MedAssist* that uses deep learning to assist physicians in the identification of patients who may be good candidates for the prescription of selective serotonin reuptake inhibitors (SSRIs). The hospital’s goal in using *MedAssist* is to identify patients who are good candidates for SSRIs, allowing the physician to prescribe expeditiously and provide patients with needed care in a more timely manner.  When a patient presents with depressive symptoms, their physician can open up *MedAssist* via an embedded application within the EHR system. Upon initiating *MedAssist*, the patient will be asked to enter their responses to the nine-question Patient Health Questionnaire (PHQ-9). *MedAssist* will then use deep learning to analyze the patient’s medical record, family history, demographics, responses to the PHQ-9, and, if available, MRI, genetic, and epigenetic information. It will output a “SSRI compatibility score,” which will be displayed to the physician and become part of the patient’s electronic medical record. | Recently, the hospital system you work for has adopted routine use of an artificial intelligence tool called *MedAssist* that uses deep learning to assist physicians in the identification of patients who may be good candidates for the prescription of ↘selective serotonin reuptake inhibitors (SSRIs). The hospital’s goal in using *MedAssist* is to identify patients who are good candidates for SSRIs, allowing the physician to prescribe expeditiously and provide patients with needed care in a more timely manner.  The hospital has provided you with the following additional information about *MedAssist*:   - MedAssist calculates an SSRI compatibility score that reflects the probability of successful completion of a full course of prescribed antidepressant treatment, taking into account the patient-specific expected side effect profile, risk of early discontinuation, and anticipated patient response. - *MedAssist* was developed by a team of AI researchers, psychiatrists, and general practitioners at a regional hospital. It was trained and tested using data sets from three hospital systems in the region with moderate sample sizes. - Neuroimaging data and non-imaging biomarkers were used to train the algorithm, a neural networks (NN) model, in which the data are abstracted in many hidden layers to predict the patient’s response to an SSRI. - The NN model behind MedAssist had an area under the curve (AUC) value of 0.83, outperforming random forest regression which had an AUC of 0.74. The model has been trained on historical patient data but not yet on newly acquired data from this hospital.   When a patient presents with depressive symptoms, their physician can open up *MedAssist* via an embedded application within the EHR system. Upon initiating *MedAssist*, the patient will be asked to enter their responses to the nine-question Patient Health Questionnaire (PHQ-9). *MedAssist* will then use deep learning to analyze the patient’s medical record, family history, demographics, responses to the PHQ-9, and, if available, MRI, genetic, and epigenetic information. It will output a “SSRI compatibility score,” which will be displayed to the physician and become part of the patient’s electronic medical record. |

↘ ↙

**RANDOMIZATION**

↙ ↘

| **Certainty of AI output - LOWER** | **Certainty of AI output - HIGHER** |
| --- | --- |
| As per hospital protocol, you take the steps related to use of *MedAssist.* It analyzes Jess’s data and outputs the following result, which your division leadership has advised that you use to help guide your decision-making:  **SSRI Compatibility score: 52.9/100**  Patient Jess Y. is in the **52nd percentile** of all patients in terms of SSRI compatibility.  Based on this score, Patient Jess Y. is likely an **AVERAGE** candidate for an SSRI prescription.  It is recommended that Patient Jess Y. is NOT prescribed an SSRI at this time. | As per hospital protocol, you take the steps related to use of *MedAssist.* It analyzes Jess’s data and outputs the following result, which your division leadership has advised that you use to help guide your decision-making:  **SSRI Compatibility score: 95.2/100**  Patient Jess Y. is in the **95th percentile** of all patients in terms of SSRI compatibility.  Based on this score, Patient Jess Y. is likely a **GOOD** candidate for an SSRI prescription.  It is recommended that Patient Jess Y. is prescribed an SSRI at this time. |

**Vignette Path #2**

| **Degree of clinical risk - LOWER** |
| --- |
| Jess Y. is a 33 year-old white female who presents to your clinic. She reports feeling extremely anxious and worried over the past three months.  Jess reports feeling anxious about her full-time job as a software engineer at a company where she has worked for six months. Although she is interested in the work, Jess reports that she frequently misses important deadlines and makes careless mistakes in her work. She reports feeling unable to focus on her work for more than a few minutes at a time, despite trying many different time management strategies.  Jess also reports increasing tension between her and her partner of five years. Previously, they agreed to start trying to have children around now, but Jess fears that she is too unreliable to be a parent. She reports that she is always late to appointments and has a hard time keeping track of household responsibilities, and worries that these tendencies will be exacerbated if she becomes a parent.  Jess reports that she received extra time for test taking in elementary and middle school, but that she never had any formal neuropsychological testing for ADHD. |

↓

**RANDOMIZATION**

↙ ↘

| **Amount of information sharing - LOWER** | **Amount of information sharing - HIGHER** |
| --- | --- |
| Recently, the hospital system you work for has adopted routine use of an artificial intelligence tool called *MedAssist* that uses deep learning to assist physicians in the identification of patients who may have attention deficit hyperactivity disorder (ADHD). The hospital’s goal in using *MedAssist* is to more accurately identify patients who require a referral for a formal ADHD assessment, in order to avoid unnecessary referrals and provide patients with needed care in a more timely manner.  When a patient presents with symptoms of ADHD, their physician can open up *MedAssist* via an embedded application within the EHR system. Upon initiating *MedAssist*, the patient will be asked to enter their responses to the six-question Adult ADHD Self-Report Scale (ASRS). *MedAssist* will then use deep learning to analyze the patient’s medical record, family history, demographics, responses to the ASRS, and, if available, MRI, genetic, and epigenetic information. It will output an “ADHD referral score,” which will be displayed to the physician and become part of the patient’s electronic medical record. | Recently, the hospital system you work for has adopted routine use of an artificial intelligence tool called *MedAssist* that uses deep learning to assist physicians in the identification of patients who may have attention deficit hyperactivity disorder (ADHD). The hospital’s goal in using *MedAssist* is to more accurately identify patients who require a referral for a formal ADHD assessment, in order to avoid unnecessary referrals and provide patients with needed care in a more timely manner.  The hospital has provided you with the following additional information about *MedAssist*:   - MedAssist calculates an ADHD referral score that reflects the probability of successful completion of a full course of prescribed antidepressant treatment, taking into account the patient-specific expected side effect profile, risk of early discontinuation, and anticipated patient response. - *MedAssist* was developed by a team of AI researchers, psychiatrists, and general practitioners at a regional hospital. It was trained and tested using data sets from three hospital systems in the region with moderate sample sizes. - Neuroimaging data and non-imaging biomarkers were used to train the algorithm, a neural networks (NN) model, in which the data are abstracted in many hidden layers to predict the patient’s likelihood of having ADHD. - The NN model behind MedAssist had an area under the curve (AUC) value of 0.83, outperforming random forest regression which had an AUC of 0.74. The model has yet to be prospectively tested.   When a patient presents with symptoms of ADHD, their physician can open up *MedAssist* via an embedded application within the EHR system. Upon initiating *MedAssist*, the patient will be asked to enter their responses to the six-question Adult ADHD Self-Report Scale (ASRS). *MedAssist* will then use deep learning to analyze the patient’s medical record, family history, demographics, responses to the ASRS, and, if available, MRI, genetic, and epigenetic information. It will output a “ADHD referral score,” which will be displayed to the physician and become part of the patient’s electronic medical record. |

↘ ↙

**RANDOMIZATION**

↙ ↘

| **Certainty of AI output - LOWER** | **Certainty of AI output - HIGHER** |
| --- | --- |
| As per hospital protocol, you take the steps related to use of *MedAssist.* It analyzes Jess’s data and outputs the following result, which your division leadership has advised that you use to help guide your decision-making:  **ADHD Referral score: 52.9/100**  Patient Jess Y. is in the **52nd percentile** of all patients in terms of requiring an ADHD referral.  Based on this score, Patient Jess Y. is likely an **AVERAGE** candidate for an ADHD referral.  It is recommended that Patient Jess Y. is NOT referred to a specialist for a formal ADHD assessment at this time. | As per hospital protocol, you take the steps related to use of *MedAssist.* It analyzes Jess’s data and outputs the following result, which your division leadership has advised that you use to help guide your decision-making:  **ADHD Referral score: 95.2/100**  Patient Jess Y. is in the **95th percentile** of all patients in terms of requiring an ADHD referral.  Based on this score, Patient Jess Y. is likely a **GOOD** candidate for an ADHD referral.  It is recommended that Patient Jess Y. is referred to a specialist for a formal ADHD assessment. |
